# Interaction between indigenous hydrocarbon-degrading bacteria in reconstituted mixtures for remediation of weathered oil in soil

**DOI:** 10.1016/j.btre.2022.e00767

**Published:** 2022-10-02

**Authors:** Nasser Al-Kaabi, Zulfa Al Disi, Mohammad A. Al-Ghouti, Theis Ivan Solling, Nabil Zouari

**Affiliations:** a Environmental Science Program, Department of Biological and Environmental Sciences, College of Arts and Sciences, Qatar University, Doha, P.O. B 2713 Qatar; b Center for Integrative Petroleum Research, KFUPM, Academic Loop Rd, Dhahran, 31261 KSA

**Keywords:** Microbial bioremediation, Weathered oil, Bioaugmentation, Biostimulation, Hydrocarbon-degrading bacteria

## Abstract

It has been demonstrated that biostimulation is necessary to investigate the interactions between indigenous bacteria and establish an approach for the bioremediation of soils contaminated with weathered oil. This was achieved by adjusting the carbon (C)/nitrogen (N)/phosphorus (P) ratio to 100/10/1 combined with the application of 0.8 mL/kg Tween-80. In addition, three indigenous bacteria isolated from the same soil were introduced solely or combined concomitantly with stimulation. Removal of n-alkanes and the ratios of n-heptadecane to pristane and n-octadecane to phytane were taken to indicate their biodegradation performance over a period of 16 weeks. One strain of *Pseudomonas aeruginosa* D7S1 improved the efficiency of the process of stimulation. However, another *Pseudomonas aeruginosa,* D5D1, inhibited the overall process when combined with other bacteria. One strain of *Bacillus licheniformis* D1D2 did not affect the process significantly. The Fourier transform infrared analysis of the residual hydrocarbons supported the conclusions pertaining to the biodegradation processes when probing the modifications in densities and stretching. The indigenous bacteria cannot mutually benefit from their metabolisms for bioremediation if augmented artificially. However, the strain *Pseudomonas. aeruginosa* D7S1 was able to perform better alone than in a consortium of indigenous bacteria.

## Highlights

· Biostimulation combined with bioaugmentation is appropriate to remediate weathered oil.
· Bioaugmentation with one strain of *P. aeruginosa* D7S1 is more performant than a consortium.
· PCA of the n-alkane, *n*-heptadecane to pristane and *n*-octadecane to phytane ratios is adequate to evaluate weathered oil removal by bacterial consortia.

## 1 Introduction

Bacteria are the most commonly used microorganisms in bioremediation. In numerous applications, either *in situ* or *ex situ*, single or mixed cultures of bacteria are used. They naturally exist as mixtures in the environment and can rapidly degrade pollutants when appropriate conditions are available. In bioremediation processes, autochthonous (indigenous) or allochthonous (introduced) microorganisms are used. Indeed, there is the possibility that the indigenous bacteria are not capable of degrading the existing pollutants because they lack appropriate metabolic pathways, their density is not sufficient, or the conditions are not suitable for the treatment. This situation mostly occurs with oil pollution in soils. In this scenario, bioaugmentation approaches using indigenous bacteria can be convenient [1]. Overcoming the chemical and physical barriers to degrade targeted pollutants using indigenous microorganisms has been the focus of a wide variety of bioaugmentation studies [2, 3, 4]. Autochthonous microorganisms should be better adapted to the environmental conditions of their habitat than allochthonous microorganisms. They can exhibit a higher potential for bioremediation and survivability [5]. Moreover, the highest biotransformation activity of the existing pollutants is exhibited by the zymogenous population of autochthonous microorganisms [6]. As a result, biotransformation or even complete mineralization of organics can occur through the overall metabolism of all existing microorganisms. The removal of oil hydrocarbons from the environment, especially from soils, is one of the proven bioremediation processes [7]. Similar processes have been applied for the biodegradation of polychlorinated biphenyls [8], polycyclic aromatic hydrocarbons (PAHs) [9,10], pesticides [11], some heavy metals [12] and even radionucleotides [13, 14]. Regarding the bioremediation of oil spills, the efficiency is determined based on the available total petroleum hydrocarbons (TPHs) at the site. Indeed, biostimulation, providing nutritional supplements or increasing the availability of pollutants and their accessibility to bacteria, was successful if appropriate microorganisms were available at the site. Nevertheless, bioaugmentation (seeding), which is described as the injection of viable bacterial cells into the natural ecosystem, facilitates the rate and/or extent of oil biodegradation [15]. However, coupling bioaugmentation to biostimulation leads to an improvement of the microenvironment [16]. This could be done by aeration in the case of aerobic microorganisms, adding nutrients if not sufficient for microorganisms, a higher density of microbial cells if needed and surfactants for hydrophobic pollutants, among others [17]. This combination is common when attempting to remediate hydrocarbon-polluted soils, as shown in temperate climates [18]. However, the climate of arid regions results in severe stress on indigenous bacteria [19], and little is known about the biodegradability of oil products [20, 21]. Such harsh conditions accelerate the weathering processes of oil components polluting the soils [22]. Therefore, the complexity of the mixtures of compounds is increased in the spilled petroleum hydrocarbons. The rate of degradation differs from one component to another depending on their chemical structure and concentration. Four main fractions characterize petroleum hydrocarbons, asphaltenes, aromatics, resins and saturates [23]. The *n*-alkanes are the most willingly degraded and are average in length (*n*-C10- *n*-C25). The longer chains of alkanes consisting of (*n*-C25- *n*-C40) are more difficult to degrade due to their low solubility in water and low bioavailability. They are considered hydrophobic solids [23]. The branched chains of cycloalkanes and alkanes take more time in the degradation process than normal alkanes. Highly condensed cycloparaffinic structures, condensed aromatics, bitumen, tars and asphaltic materials are the hardest to biodegrade and have high boiling points. After oil degradation, the residual product can be regarded as a humic substance [23]. Oil TPHs include molecules of *n*-C2- *n*-C40). They can be further separated into petroleum-range organics (*n*-C2- *n*-C5) and diesel-range organics (DRO) (*n*-C6- *n*-C40) [24, 25]. During the biodegradation of hydrocarbons, the main question that arises is how microbial cells interact with pollutants. Indeed, bacteria that are biodegraders of oil components should also produce biosurfactants to increase the bioavailability of hydrophobic oil organics [26, 27]. However, the microorganisms of the soil do not necessarily possess the ability to metabolize pollutants if not exposed to contaminating compounds. Nevertheless, once these microorganisms have been exposed to pollutants, they are also able to adapt by acquiring metabolic capabilities by vertical or horizontal recruitment of genes [28, 29]. Recruitment of new metabolic pathways, present in the genome but not expressed, may be done by events of mutation, genetic rearrangement or transposition referring to vertical recruitment [28, 30]. Microorganisms can also acquire catabolic gene clusters via mobile elements transferred from a donor to a receiving host, referring to horizontal transfer [31]. In bacteria, transfer phenomena are best known through the combination of plasmids, transformation or transduction [32]. These phenomena of horizontal transfers improve the catabolic capabilities of bacteria in many ways, including expansion of their substrate range, allowing their adaptation with other members of the microbial populations to other pollutants [33].

Processes of biodegradation of complex mixtures of hydrocarbons could require cooperation between more than one species together [34]. This is true particularly in types of pollution that are made by various compounds, such as petroleum or crude oil, and desired for complete mineralization to H_2_O and CO_2_ [35]. Individual species of microorganisms may metabolize a limited range of hydrocarbons. Assembling mixed microbial populations with a high range of enzymatic capacities further increases the extent and rate of hydrocarbon biodegradation [36]. Populations of microorganisms that consist of various strains belonging to different genera have been observed and detected in water or soils contaminated with petroleum [37, 38]. Each genus strain or a different strain of a genus in a microbial community plays a significant role in the biotransformation, and the presence of other species or strains, when the energy source is limited, may limit their presence and survival, especially with complex substrates [39]. As such, the metabolic diversity of bacteria that degrade hydrocarbons facilitates the process of degradation but is also the source of failure of some bioaugmentation applications [40]. This especially occurs with weathered oil. The gap in the knowledge is about the behavior of reconstituted mixtures (consortia) of indigenous bacteria in a weathered soil polluted with oil. Indeed, intermediates of metabolic pathways can act as substrates or inhibitors for other bacteria [41]. The weathered oily soil from the Dukhan area in Qatar represents a model of soil in which such a question may be answered at the microbial-behavior level, rather than at the metabolic level. The objectives of the current work are to (i) assess self-purification by biostimulation of the highly weathered oily soil of the Dukhan dumping site, (ii) investigate the potential of preselected isolated indigenous bacterial strains (one *Bacillus licheniformis* and two *Pseudomonas aeruginosa)* individually bioaugmented or in mixtures of two or three in the biostimulated biopiles, and (iii) evaluate the changes in soil organic composition by Fourier transform infrared (FTIR) analysis to make a decision about the appropriate mode of biostimulation/bioaugmentation for the remediation of highly weathered oily soils.

## 2 Material and methods

### 2.1 Soil sampling, homogenization, and characteristics

Soil samples were obtained by composite sampling from the Dukhan oil waste dumping site. The samples were then sieved with 2 mm pore size sieves and manually mixed to reach homogeneity. The average soil moisture was 6.5 wt. %. This was determined by drying soils at 50 °C for 5 days (stable weight). The soil contained 23% carbon of the total dry matter with amounts of nitrogen and phosphorus (below the detection level) using a carbon, hydrogen, nitrogen, and oxygen (CHNO) analyzer (FLASH 2000-Thermo Fisher Scientific). The Diesel Range Organics (DRO) and PAHs in the soils were measured using GC-FID and gas chromatography–mass spectrometry (GC‒MS), and their contents in the control biopile were 3067 ppm and 403 ppb, respectively. All analyses were performed in triplicate for proper statistical representation. The detailed procedures of the abovementioned methods are described by Oualha et al. [42].

### 2.2 Bioremediation in biopiles

Each biopile contained 712 g of soil. All biopiles were carried out in triplicate under the same conditions. Moisture in the soil samples was adjusted from 6.5% initially to 10% by mixing 27 mL distilled water for each 685 g of soil. The biopiles were incubated in an oven set at 30°C. For the weekly analyses, 5 g was sampled from each biopile after rigorous manual homogenization for 5 min. The methods and the related analyses are described by Oualha et al. [42].

### 2.3 Biopiles for biostimulation

Biostimulation was performed as described by Oualha et al. [42] with adjustment of the C/N/P ratios in the soils using ammonium nitrate as a nitrogen source containing 46.7% nitrogen and potassium phosphate as a phosphorus source containing 22% phosphorus. The corresponding C/N/P was 100/10/1 [43]. Twee-80 kg^−1^ (0.8 mL) was added as a chemical surfactant.

### 2.4 Biopiles for bioaugmentation

Three indigenous bacterial strains, D1D2 (*Bacillus licheniformis*), D5D1 (*Pseudomonas aeruginosa*) and D7S1 (*Pseudomonas aeruginosa*), were previously isolated from the same dumping site in the Dukhan, and classified as highly hydrocarbon-degrading strains [44]. In total, and eight different biopiles were tested in triplicate using single or mixed bacterial strains, as shown in Table 1.

**Table 1:** Single and mixed indigenous bacterial strains used in bioaugmentation/biostimulation remediation of soil from the Dukhan dumping site.

	**Indigenous bacterial strain**		
**Biopile #**	**D1D2 (*Bacillus) licheniformis*)**	**D5D1 (*Pseudomonas) aeruginosa*)**	**D7S1(*Pseudomonas) aeruginosa*)**
**T1**	X	-	**-**
**T3**	-	X	**-**
**T5**	-	-	**X**
**T7**	X	X	**-**
**T9**	X	-	**X**
**T11**	-	X	**X**
**T13**	X	X	**X**
**T15 (control)**	**-**	**-**	**-**

Bioaugmentation was performed by inoculating the biopiles with the bacterial strains at an initial cell count of 2 × 10^4^ CFU g^−1^ soil at the beginning of the incubation. If only one strain was used, its initial count was 2 × 10^4^ CFU g^−1^soil at the time of inoculation. If a consortium of three strains is used, the total cell count of all the strains was also 2 × 10^4^ CFU g^−1^ soil, which means that  each strain was inoculated with a respective cell count of the third of inoculum size (0.667 × 10^4^ CFU g^−1^ soil). At such conditions, it would be possible to compare the performance of an individual strain to the reconstituted consortium.

The inoculum was prepared from separate liquid cultures prepared by inoculating 20 mL Minimum Salts Medium (MSM) as described by [40]), with a strain colony supplemented with 10% diesel and incubated for one week in a shaker set at 200 rpm and 30°C. The cultures were then centrifuged (5,000 rpm for 5 min), and the pellets were harvested, washed twice with MSM, and suspended in 5 mL MSM as reported by Oualha et al. [42]. Cell counts of the suspensions were determined as CFU on LB solid medium. The necessary volume for bioaugmentation was calculated accordingly. The control (T15) is performed with biostimulation only. In addition, another control was also performed without stimulation or augmentation (not shown). However, the indigenous bacteria in this control were not able to grow and thus could not remove hydrocarbons (results not shown).

### 2.5 Determination of the total bacterial cells (CFU determination)

The cell count in the inoculum was determined as colony forming units (CFU) by spreading 100 μL of serial dilutions on Luria-Bertani solid plates and incubating at 30°C. This rich medium was used because it allowed fast growth and formation of colonies after overnight incubation at 30 ^°^C, as reported by Oualha et al. [42]. One gram of soil was sampled from each biopile following a 5 min manual homogenization and suspended in 5 mL MSM. Then, CFUs in the soil were determined.

### 2.6 GC-FID and GC‒MS analyses

The *n*-alkane range was analyzed by GC-FID as detailed by Al-Kaabi et al. [23] and Oualha et al. [42]. Gas chromatography was periodically performed for *n*-C17 to *n*-C36 hydrocarbon determination to evaluate the efficiency of their removal by the bacterial isolates or the consortia. Analysis of hydrocarbons by GC‒MS was performed during the incubation of the biopiles. Changes over time of *n*-alkanes from *n*-C17 to *n*-C36 in the soil were monitored in each biopile.

### 2.7 Fourier transform infrared (FTIR) analysis

The soil samples from each biopile and potassium bromide (KBr) were first dried in an oven set at 50 °C for 48 h and then manually ground using a spatula. FTIR analysis was performed with a mixture of 20 mg of the soil and 200 mg KBr in a 13 mm disk at a pressure of 7 tons for 2 min using an FTIR PERKINELMER (Spectrum BXII). Transmittance spectra were generated with wavenumbers in the 400-4000 cm^−1^ range.

### 2.8 Statistical analysis

The results were obtained in triplicate and statistically analyzed by analysis of variance (ANOVA) at a 95% confidence level.

## 3 Results

### 3.1 Bioremediation of weathered polluted soils by biostimulation in biopiles

**Fig. 1:**
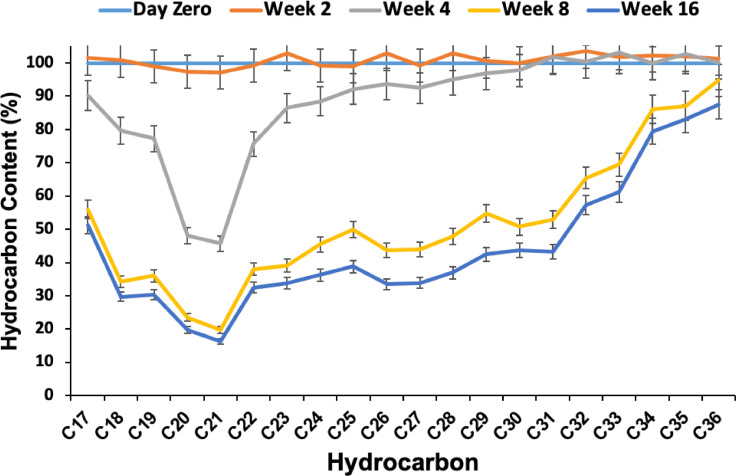
Hydrocarbon removal from oily soil of the Dukhan dumping area using biostimulation, with adjustment of C/N/P to 100/10/1 and adding 0.8 ml Twee-80/kg. The corresponding biopile code is T15 in Table 1.

The removal of hydrocarbons from the oily soil of the Dukhan dumping area was monitored in biopiles, performed with biostimulation only (Biopiles#15 in Table 1). Fig. 1 shows the results of the removal of *n*-alkanes over the incubation period of bioremediation by biostimulation. Biodegradation started effectively after 4 weeks with molecules of lower molecular weights (from *n*-C-17 to *n*-C-29), and the highest removal was marked with *n*-C20- *n*-C21. At week 8, most of the alkanes were removed, and indigenous bacteria developed an almost linear decrease in activity with the increase in the molecular weight of hydrocarbons. The activity beyond the 8^th^ week was lower. At 16 weeks, more than 60% of each alkane of less than *n*-C32 was registered. The highest removal of up to 85% of *n*-alkanes obtained by biostimulation of the indigenous bacteria was recorded in the 16^th^ week.

Regarding the bacterial growth in the biopiles, the CFU of the biopile soil was determined during the incubation, knowing that the initial CFUs were approximately 3 × 10^2^ CFU g^−1^ in the noninoculated soil. The determined CFUs corresponded to the colonies formed by all the strains on LB solid medium. It was not possible to determine the CFU of each separate strain. The CFUs were 4 × 10^4^ CFU g^−1^, 9 × 10^4^ CFU g^−1^ and 2 × 10^5^ CFU g^−1^ at weeks 4, 8 and 16, respectively. This result shows that the stimulation of growth of the indigenous bacteria can be achieved by adjusting the C/N/P ratio to 100/10/1 with the addition of 0.8 mL Tween-80 kg^−1^. The bacteria showed a high adaptation to degrade the residual hydrocarbons in the weathered soil.

### 3.2 Efficiency of bioaugmentation using a single indigenous bacterial strain in oily soil of the Dukhan dumping site

**Fig. 2:**
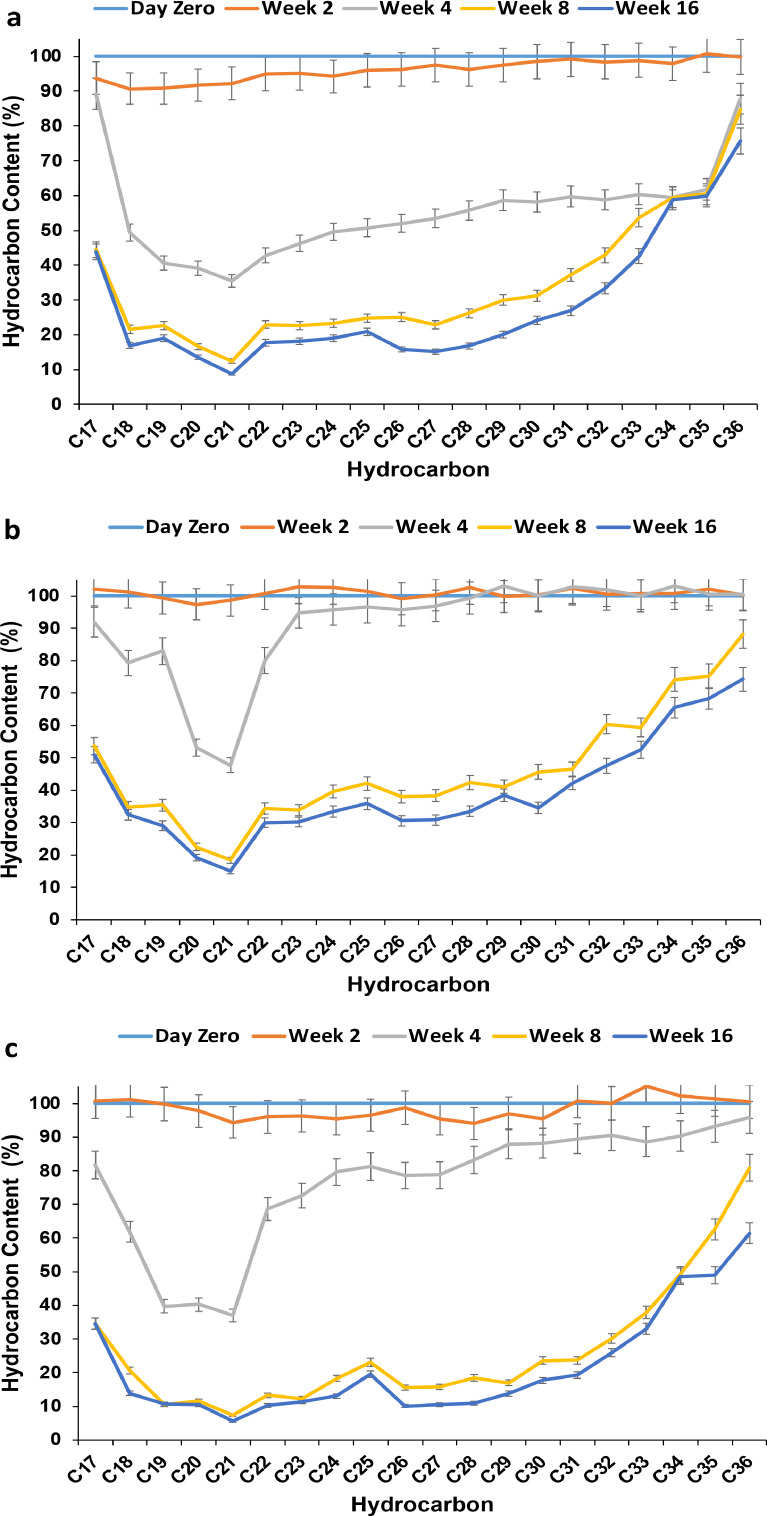
Removal of *n*-alkanes by biostimulation/bioaugmentation using the indigenous bacteria **a)***B. licheniformis* D1D2, **b)***P. aeruginosa* D5D1 and **c)***P. aeruginosa* D7S1.

To study the efficiency of bioaugmentation coupled to biostimulation on the removal of *n*-alkanes, the isolates D1D2, D5D1, and D7S1 initially isolated from the same polluted soil were introduced individually into the biopiles (T1, T3 and T5, respectively). The results are shown in Fig. 2. Using *B. licheniformis* D1D2 or *P. aeruginosa* D7S1, degradation of *n*-C17 to *n*-C21 hydrocarbons started effectively on week 2, whereas using the strain *P. aeruginosa* D5D1, it started on week 4, as was done for biostimulation alone. It seems that D1D2 and D7S1 could reduce the lag phase of the bioremediation on the fraction of *n*-C17 to *n*-C21 hydrocarbons, while D5D1 did not change the dynamics of removal compared to biostimulation alone. The highest removal rate of *n*-alkanes was observed with *n*-C21 after week 16, reaching up to 95%, 92%, and 85% with D1D2, D5D1, and D7S1, respectively. Alkanes with lower molecular weights underwent a higher degradation compared to those with higher molecular weights. After 16 weeks of incubation, the removal efficiency of *n-*C36 was 39% with D7S1, while it was much lower with D5D1 (25%) and D1D2 (24%).

The efficiency of the strains was also translated into growth by increasing their respective CFUs in the soil. The determined CFUs corresponded to the colonies formed on LB solid medium by the strains existing in the soil. It was not possible to determine the CFU of each separate strain. Regarding the bacterial growth in the biopiles, the CFU in the biopile soils was determined during the incubation, knowing that the initial CFUs were approximately 2 × 10^4^ CFU g^−1^. The CFUs reported in Table 2 show that the growth of bacteria in the augmented/stimulated biopiles is much higher during the incubation than that obtained with stimulated growth only.

**Table 2:** Growth evaluation of the inoculated strains through CFU determination

**Biopile #**	**Inoculated strain**	**CFU (10^5^ CFU g^−1^) after:**		
		**4 weeks**	**8 weeks**	**16 weeks**
T1	*B. licheniformis* (D1D2)	29 ± 1	35 ± 2	37 ± 1
T3	*P. aeruginosa* (D5D1)	33 ± 1	36 ± 2	39± 1
T5	*P. aeruginosa* (D7S1)	42 ± 1	56 ± 2	57 ± 1

### 3.3 Performance of biostimulation/bioaugmentation using reconstituted consortia in oily soils of the Dukhan dumping site

**Fig. 3:**
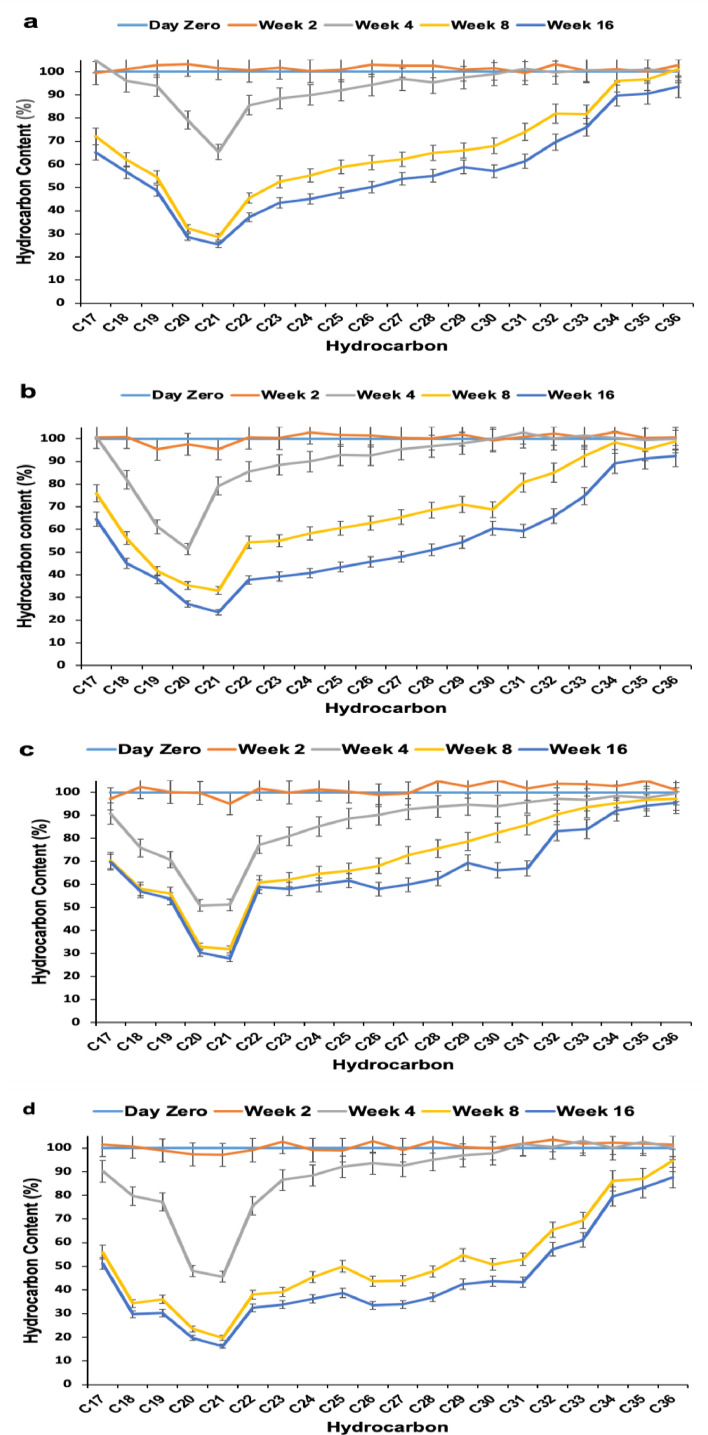
Effect of bioaugmentation on the degradation of hydrocarbon compounds using a combination of studied isolates: a) T7 (D1D2+D5D1), b) T9 (D1D2+D7S1), c) T11 (D5D1+D7S1) and d) T13 (D1D2+D5D1+D7S1).

The efficiency of reconstituted bacterial consortia using the indigenous bacterial strains isolated from Dukhan soil was investigated in stimulated biopiles. Combinations of two strains or all three strains together were evaluated (Fig. 3).

The results in Fig. 3 show the removal of each *n*-alkane using the consortia in biopiles. It is clear that the removal of *n*-alkanes was pronounced on week 4 with hydrocarbons having low molecular weights (less than *n*-C25). However, with the combinations D1D2/D5D1 and D1D2/D5D1/D7S1, the removal was low during the first two weeks on *n*-C17 to *n*-C19 and was generally lower on all *n*-alkanes compared to the single strains (Fig. 2). The combination of D5D1 to D1D2 or to D7S1 or to D1D2/D7S1 reduced the performance of the individually used strains. The highest removal efficiencies of *n*-alkanes were observed in week 16, with up to 74%, 74%, 76%, and 70% of *n-*C21 using the combinations D1D2/D7S1, D5D1/D7S1, D1D2/D5D1, and D1D2/D5D1/D7S1, respectively. These performances are almost 20% lower than those with individual strains, as shown in Fig. 2. The same conclusion is clear with the removal efficiencies of *n-*C36, which were 25%, 25%, 24%, and 16%, respectively.

The CFUs determined in the biopiles performed with the combined strains after 4, 8 and 16 weeks of incubation (Table 3) show that the combination of the 3 strains led to the highest CFU during all the periods of incubation. However, the CFUs obtained with single strains (Table 2) were higher. It seems that the interaction between the strains inhibited their growth to a certain extent and that the competition in the substrate uptake between the strains to induce growth was unfavored in the combination.

**Table 3:** Growth evaluation of the inoculated strains through CFU determination

**Biopile #**	**Inoculated strain**	**CFU (10^5^ CFU g^−1^) after:**		
		**4 weeks**	**8 weeks**	**16 weeks**
T7	D1D2 + D5D1	21 ± 1	29 ± 2	30 ± 2
T9	D1D2 + D7S1	28 ± 2	31± 2	33± 2
T11	D5D1+ D7S1	24 ± 1	28± 2	31± 2
T13	D1D2 + D5D1 +D7S1	32 ± 2	36 ± 2	37 ±2

### 3.4 Performance and rate of biostimulation/bioaugmentation for the bioremediation of weathered oily soil

**Fig. 4:**
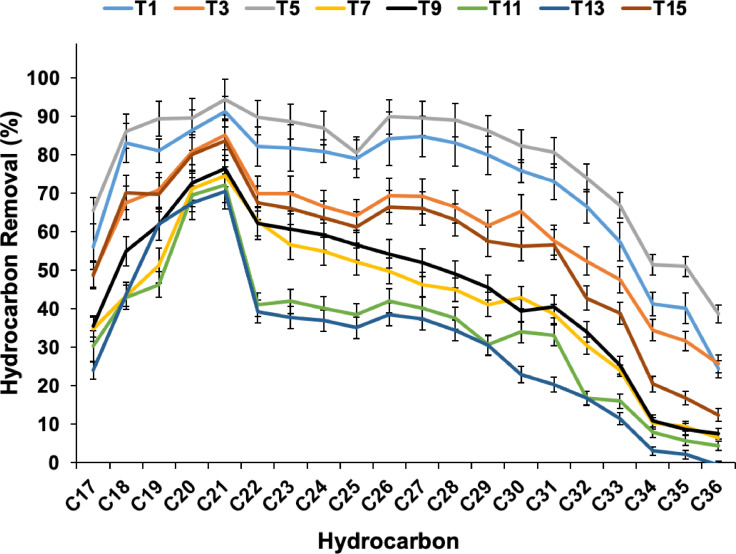
Efficiency of bioaugmentation using single or reconstituted consortia of the indigenous bacteria compared to biostimulation of the indigenous bacteria in oily soil of the Dukhan dumping site after 16 weeks of incubation.

To compare the effect of biostimulation/bioaugmentation using single indigenous isolates or their mixtures (consortia) on the rate and efficiency of degradation of hydrocarbons, their corresponding removal profiles were compared after 16 weeks of treatment (Fig. 4).

The best removal profile was obtained with bioaugmentation using isolate D7S1 (biopile#T5). The isolate D1D2 (Biopile#T1) performed slightly worse than the isolate D7S1 (biopile#T5). Biostimulated biopile#T15, used as a control for bioaugmentation, showed similar results to that of the biostimulated/bioaugmented biopile#T3 performed using the strain D5D1. This means that bioaugmentation with the strain *P. aeruginosa* D5D1 did not improve the overall performance of the existing bacteria in the soil. Surprisingly, bioaugmentation using the mixed bacterial isolates (biopile#T13) was less efficient than biostimulation without bioaugmentation (biopile#T15). Indeed, augmentation using a combination of D1D2 to D5D1 or to D7S1 was less efficient than just biostimulation and much less efficient than bioaugmentation using D1D2 alone. Similar conclusions can be drawn regarding isolate D7S1 (biopile#T5), which was much more efficient than all the other combinations. The combination of D5D1 to D7S1 or the reconstituted consortium with the three isolates together significantly decreased the performance of the removal of hydrocarbons. These results clearly show that mixing the isolated indigenous bacteria in bioaugmentation approaches was not beneficial for the treatment of the weathered oily soils at the Dukhan site. However, the strain *P. aeruginosa* D7S1 was able to perform better results alone than when mixed with the other indigenous bacteria.

### 3.5 Evaluation of the performance of biostimulation/bioaugmentation on *n*-alkanes (*n*-C17-*n*-C19) and their branched homologs

**Fig. 5:**
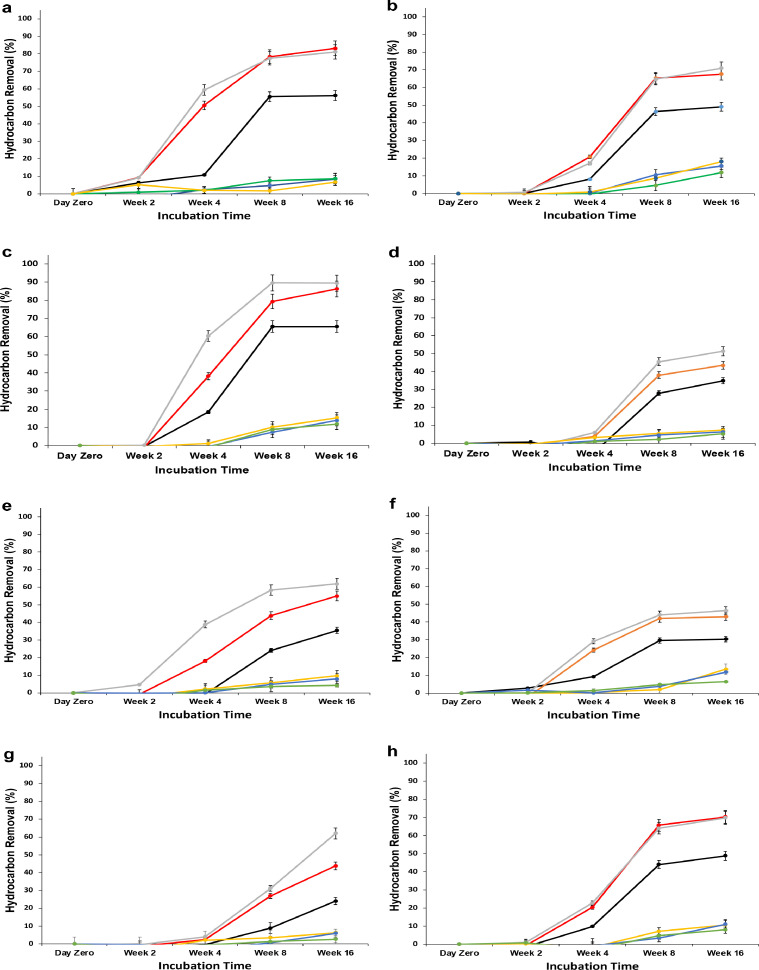
Evaluation of the performance of the biostimulation/bioaugmentation treatments using indigenous bacteria on *n*-C17-n-C19 alkanes and their branched homologs: a) T1, b) T3, c) T5, d) T7, e) T9, f) T11, g) 13, and h) T15 Biostimulation (no augmentation).

Since the ratios of *n*-heptadecane (*n*-C17) to pristane (Pr) and *n*-octadecane (*n*-C18) to phytane (Ph) are indicative of biodegradation performance and their removal at different rates is a result of the specific mechanism of biodegradation, the removal of *n*-alkanes in the range *n*-C17 to *n*-C19 and their branched homologs were evaluated during the treatment in all performed biopiles (Fig. 5). The bioaugmentation by a single bacterial strain reduced the hydrocarbon concentration to less than 15% at week 16. The depletion of *n*-C17 to *n*-C19 as well as branched homologs could be attributed to biodegradation. With all combinations of biostimulation/bioaugmentation, the removal of C21, phytane, and pristane never exceeded 10% (residual contents were always near 90%). For the branched alkanes in all biopiles, removal efficiencies were below 10% at week 8, while they further improved after 16 weeks of incubation to 13% for D1D2, 18% for D5D1, and 15% for D7S1, while they remained at 11% for the control (biostimulation only). For all combinations of bacterial strains, the branched alkane reduction was below 10%. As seen for *n*-C17-*n*-C36, both *n*-alkanes and branched alkanes were generally degraded at a higher rate with shorter chain length.

### 3.6 Investigation of changes in hydrocarbon composition using FTIR analyses

**Fig. 6:**
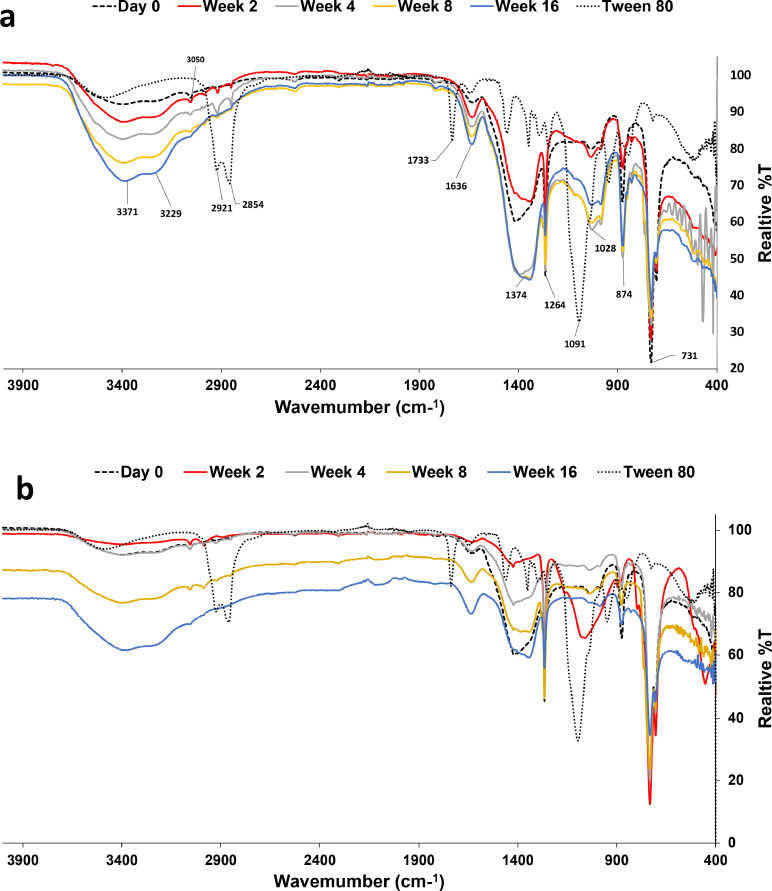

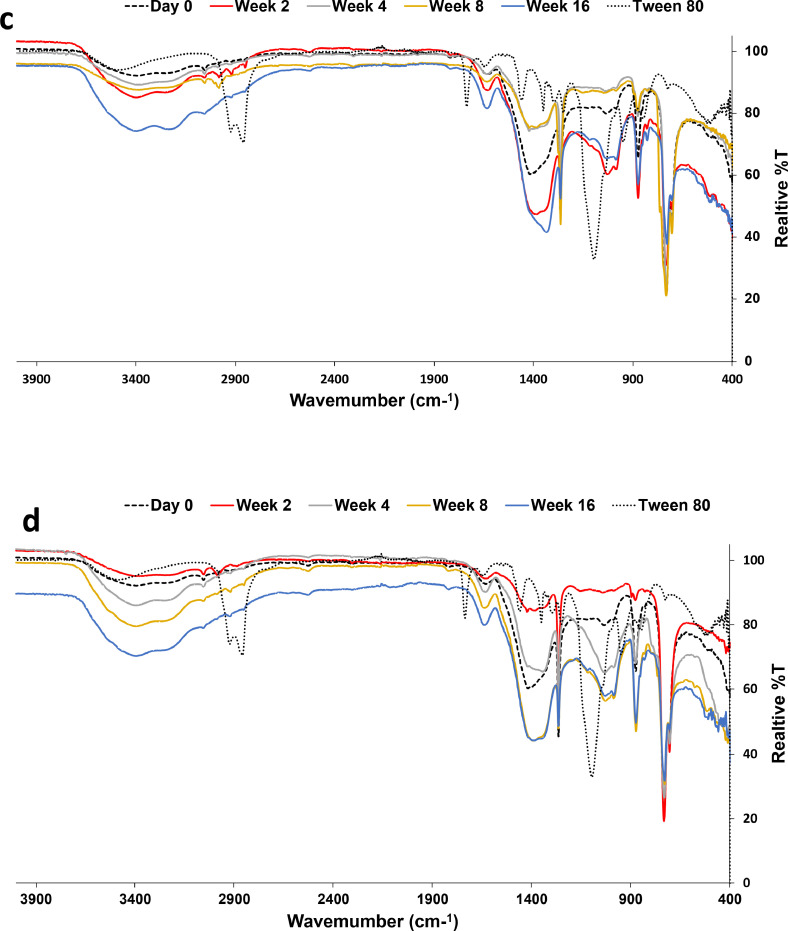

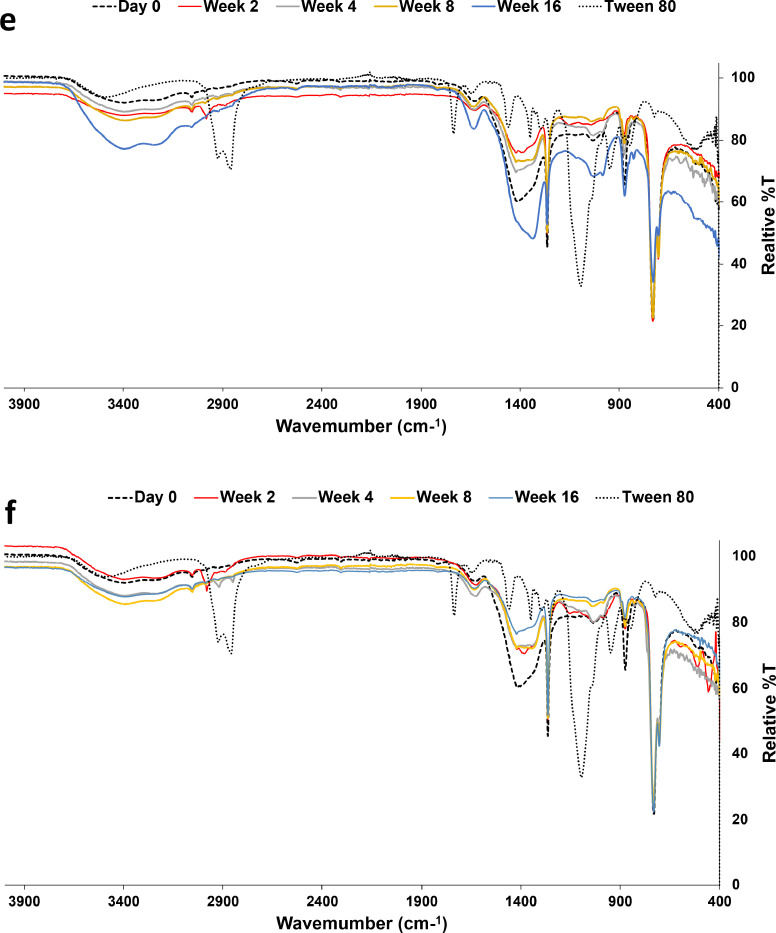

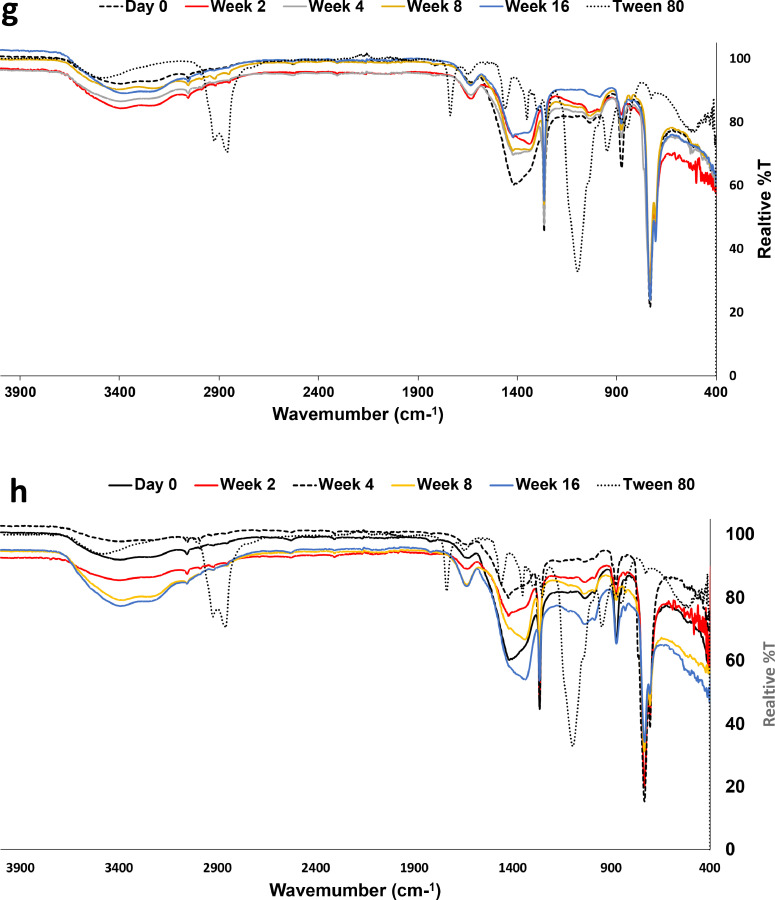
FTIR spectra of the soils with a) T1, b) T3, c) T5, d) T7, e) T9, f) T11, g) 13, and h) T15 Biostimulation (no augmentation).

The FTIR analyses are shown in Fig. 6 The FTIR spectrum (Fig. 6a) of the Dukhan soil before any treatment (stimulation without augmentation) shows aromatic C−H stretching vibrations at approximately 3050 cm^−1^ (Minejima et al., 2002) and peaks of aliphatic hydrocarbons (-CH_2_ and -CH_3_) stretching at 2987 cm^−1^ [45]. The O-H stretching vibrations are responsible for the broad peaks estimated at 3200–35450 cm^−1^ [46]. The changes in the structural properties of the Dukhan soil after the addition of the nonionic surfactant Tween-80 resulted in the formation of the major peak located between 1000–1100 cm^−1^, associated with S=O or C-O stretching vibrations, as shown in Fig. 6b. These findings are in agreement with the results of Feng et al. (2006), who showed that the addition of Tween 80 to diesel oil enhances biodesulfurization. The absorptions in the 680–730 cm^−1^ range represent the C-H aromatic groups undergoing out-of-plane deformation stretching [47]. The band at 1630 cm^−1^ is associated with carbonyl C=O [47].

The peaks located at 2834 cm^−1,^ 2914 cm^−1^ and 2987 cm^−^1, representing -CH_2_ and -CH_3_ stretching, disappeared during the incubation. However, their removal was performed at different rates; starting week 4 in the biostimulation treatment and the decrease in the band at 704 cm^−1^ (alkene =C-H), this means that bacteria start breaking down the alkenes to transform them to alkanes, resulting in the elimination of the 2987 cm^−1^ peak. In addition, with the reduction of the band at 730 cm^−1^, the alkane concentration starts to decrease due to degradation, as shown by Kumar et al. [48]. The FTIR spectra also showed a decrease in the intensity of the aromatic C-H stretching bands ranging between 3000 and 3100 cm^−1^ at weeks 2, 4, 8, and 16. Stretching vibrations at approximately 3200-3550 cm^−1^ were increased in O-H at weeks 2, 4, 8, and 16, generated by the microbial oxidation process. The alkene signal at 731 cm^−1^ starts to decrease in intensity until week 16, which is an indication of biodegradation. In addition, an increase in the intensity of the stretching vibrations of C-O was illustrated in the FTIR spectra at 1034 cm^−1^.

## 4 Discussion

The soil samples were collected from a three-year abandoned site of solid and liquid petroleum wastes. The average soil moisture of the samples was 6.5 wt.%. This was not surprising for soils sampled from an arid region. However, this moisture is not adequate for microbial growth. Previous work on the same soil [23, 49] showed that heavily weathered oil solidified and enclosed relatively unweathered oil inside the tars. This situation thus prevented further degradation of the interior oil, although three hydrocarbon-degrading bacteria were isolated from such soils after grinding and sieving the soils using 2 mm sieves. This demonstrates that degradation of oil is a very slow process and that physical and chemical weathering processes are more pronounced in this soil than in biological soil. In this study, moisture was adjusted to 10% by adding water as previously reported for the same soil [42]. Our focus was on the evaluation of hydrocarbon removal under several conditions, mainly by biological processes. Hydrocarbons existing in the soil were shown to be highly weathered when exposed to harsh conditions for more than three years [40, 23]. These results also showed that most of the hydrocarbons were highly adsorbed and stabilized in the soil particles. Under harsh weather conditions, their adsorption might increase in tar balls, which may affect the hydrocarbon structures [50]. Consequently, analysis of *n*-C17 to *n*-C36 hydrocarbons was performed periodically for 16 weeks to evaluate the efficiency of their removal by the bacterial isolates or consortia. In fact, the period of 16 weeks for the treatment may be considered acceptable for an innovative bioremediation approach at the status of the soil. In the literature, longer periods of treatment were reported to have a moderate impact on weathered oil components. Moreover, the results were highly similar in the triplicates of the performed biopiles as analyzed by ANOVA at the 95% confidence level (p values > 0.05). This statistical analysis is a necessary tool to confirm the significance of the differences in the alkane profiles under diverse bioaugmentation-biostimulation conditions, considering that many experimental artifacts in the biopiling technique may occur at a small scale. Changes over time of *n*-alkanes from *n*-C17 to *n*-C36 in the soil were monitored in each biopile, conducted using single or mixed bacteria. The objective was to elucidate the interaction between the indigenous bacteria in the case of highly weathered hydrocarbons. Many cases of bioaugmentation failure in similar regions characterized by high weathering have been reported. Adequate indigenous or exogenous bacteria should be highly adapted to soil composition but also able to cooperate with the rest of the indigenous microorganisms.

Under biostimulation conditions, a long lag phase was necessary for the indigenous bacterial communities to adapt and remove up to 85% of *n*-alkanes in the 16^th^ week, starting with the easiest to the least biodegradable substrates. Indigenous bacteria already in the soil were responsible for such removal since the nonstimulated soil did not show any changes in *n*-alkanes at 10% moisture for periods exceeding 12 months (results not shown). Under such conditions, the abiotic factors of the soil should similarly influence the rate of biodegradation in all biopiles. They include soil salinity, pH, moisture, oxygen, redox potential, and nutrient availability. The resistance of hydrocarbons to degradation is also due to their composition, solubility, and interaction between all the soil components. However, the effectiveness of bioremediation is additionally dependent on the potential of the existing bacteria to make hydrocarbons more available and susceptible to degradation.

The combination of biostimulation and bioaugmentation using the most highly adapted indigenous bacterial strains previously isolated showed that *B. licheniformis* D1D2 and *P. aeruginosa* D7S1 reduced the lag phase of bioremediation, while *P. aeruginosa* D5D1 did not change the dynamics of removal compared to biostimulation alone. Although the removal of *n*-C21 after 16 weeks ranged from 85% to 95% and hydrocarbons with lower molecular weights underwent a higher removal rate, the removal efficiency at higher molecular weights for *n-*C36 was 39% with *P. aeruginosa* D7S1, which was higher than that with *P. aeruginosa* D5D1 and *B. licheniformis* D1D2. As an overall observation, *P. aeruginosa* D7S1 can be considered the most active on *n*-alkanes in the period over 16 weeks. The isolate *B. licheniformis* D1D2 was slightly less performant than the isolate *P. aeruginosa* D7S1, while *P. aeruginosa* D5D1 seems not to be effective for bioaugmentation using a single strain. Indeed, the biostimulated biopile used as a control for bioaugmentation showed similar results to that of the biostimulated/bioaugmented biopile performed using the strain *P. aeruginosa* D5D1. It is worth to note that the three strains were selected based on their performance in the removal (%) of TPHs in liquid cultures as reported in our previous studies. Indeed, the strain *B. licheniformis* (D1D2) exhibited the highest TPH removal of 48.1 ± 0.9 %, followed by the strain *P. aeruginosa* (D5D1) with TPH removal %of 42 ± 1 % and *P. aeruginosa* (D7S1) with TPH removal of 27.0 ± 0.8 % [44].

To elucidate the consequence of the interactions between the three indigenous bacterial strains, reconstituted consortia were inoculated into the biopiles. Surprisingly, bioaugmentation using the three bacterial strains mixed in the same biopile was less efficient than biostimulation without bioaugmentation. However, the combination of *B. licheniformis* D1D2 with *P. aeruginosa* D5D1 or with *P. aeruginosa* D7S1 was less efficient than biostimulation alone and much less efficient than bioaugmentation using *B. licheniformis* D1D2 alone. Similar conclusions can be drawn regarding isolate D7S1, which was much more efficient than all the other combinations. The combination of *P. aeruginosa* D5D1 with *P. aeruginosa* D7S1 or the reconstituted consortium with the three isolates together greatly decreased the performance of the removal of hydrocarbons. These results clearly show that mixing the isolated indigenous bacteria in bioaugmentation approaches was not beneficial for the treatment of the weathered oily soils at the Dukhan site. It seems that bacteria were inhibited through their respective activities. Compared to their respective performance in removing hydrocarbons obtained in separate cultures, the profile of removal was maintained, but the removal rates and efficiencies were much reduced. Thus, it can be concluded that the indigenous bacteria cannot mutually benefit from their metabolism for growth if augmented artificially but are also inhibited to some extent. However, the strain *P. aeruginosa* D7S1 was able to perform better alone than when mixed with the other indigenous bacteria.

It is expected that the most susceptible oil components to biodegradation are *n*-alkanes and isoprenoid aliphatic alkanes [51, 52]. The ratios of *n*-heptadecane (*n*-C17) to pristane (Pr) and *n*-octadecane (*n*-C18) to phytane (Ph) indicate biodegradation performance, assuming that the isoprenoid hydrocarbon pristane (*n*-C19) and phytane (*n*-C20) volatility is similar to *n*-C17 and *n*-C18 and that their disappearance at different rates is a result of a mechanism of biodegradation rather than through simple evaporation [53]. Isoprenoid susceptibility to microbial degradation is lower than that to *n*-alkanes of similar molecular weight. In addition, their rate of evaporation and degradation tends to decrease, depending on their degree of alkylation [54]. Bioaugmentation by a single strain (D1D2, D5D1, and D7S1) reduced their concentration to less than 15% at week 16. The depletion of *n*-C17 to *n*-C19 as well as branched homologs could be attributed to their biodegradation. With all combinations of biostimulation/bioaugmentation, the removal of iC21, phytane, and pristane never exceeded 10% (residual contents were always near 90%). For all combinations of bacterial strains, the branched alkane reduction was below 10%. As seen for *n*-C17-*n*-C36, both *n*-alkanes and branched alkanes were generally degraded at a higher rate with shorter chain length. These results are analogous to those of other studies [54].

The FTIR spectra obtained in soils treated with bioaugmentation using a single strain or combined one showed similar absorption bands to those in the untreated soil or biostimulation treatment over the same periods of incubation. However, the spectra have varying relative densities. The microbial process induced an extensive response on the carbonyl C=O located at 1630 cm^−1^ through hydrocarbon oxidation continuously during the period of 16 weeks, but with variable rates among the treatments. As such, bacteria caused an oxidative alteration in the macromolecular structure resulting in the formation of oxygenated functional groups after biodegradation. The -CH_2_ and -CH_3_ stretching disappear during the incubation. In addition, an increase in the intensity of the stretching vibrations of C-O indicated some kinds of biodegradation, as shown by Kumar et al. [48]. Sequentially, there was the disappearance of some aromatic nuclei peaks because of degradation.

The study also confirmed that the susceptibility to microbial degradation is lower in high-molecular-weight *n*-alkanes than in lower-molecular-weight *n*-alkanes, which illustrates the high susceptibility previously reported by Brzeszcz & Kaszycki [29] for unweathered hydrocarbons.

Future studies on soil microbial communities can be performed using metagenomics. This would include taxonomic and functional gene composition as well as for potential new biocatalysts and enzymes [55]. Genome-resolved metagenomic techniques can be utilized to characterize the changes in microbial communities during the bioremediation (biostimulation/bioaugmentation) processes [56]. Moreover, the 16S rRNA sequences of bacterial communities can be used to predict the functional composition of the metagenome of the soils subjected to bioaugmentation [57].

## 5 Conclusion

Modern approaches to bioremediation should consider the functional biodiversity of microorganisms, which is useful for implementing them in applications. In our approach, hydrocarbon-degrading bacteria would work as a factory with high interaction with the microenvironment. This is the basic concept of a cell factory. Our strategy of isolation was highly effective in providing evidence on the microbial and metabolic variabilities. Local microbial strains exhibited interesting biological activity due to their adaptation to prolonged extreme temperatures and desiccation. The most valuable observation of the current study is that it should be expected that each site polluted with oil components should be bioremediated by intrinsic hydrocarbon-degrading bacteria to overcome long adaptation periods and provide the appropriate metabolic activities to interact specifically with the existing pollutants.

The role of soil physicochemical characteristics, the conditions and their interactions between soil pollutants and bacteria were investigated to implement the best bioremediation strategies regarding required nutrients to support bioaugmentation-biostimulation approaches in Qatar. Our results showed that a mixture of isolated indigenous bacteria caused a decrease in the removal efficiency of hydrocarbons and thus was not beneficial for the biostimulation/bioaugmentation approaches in the weathered oily soils. It seemed that bacteria were inhibited through their respective activities. Thus, we can conclude that bacteria may not be able to mutually benefit from the metabolism of each other for growth but rather inhibit growth. In this study, we showed that bioremediation by bioaugmentation/stimulation of weathered oil-contaminated soils under harsh conditions is possible, but if suitably selected indigenous bacteria are used with an appropriate screening program. Bioaugmentation must use native bacteria as they are, or at least can become, highly adapted particularly if the oil hydrocarbons are weathered. These results can explain the failure of some bioaugmentation applications, especially with weathered oil. The gap in the knowledge is about the behavior of reconstituted mixtures (consortia) of indigenous bacteria in a weathered oily soil. Indeed, intermediates of metabolic pathways could play the role of substrates or inhibitors for other bacteria. In Dukhan soils, a single bacterium augmented with the stimulation was shown to be efficient.

## Declaration of Competing Interest

The authors declare that they have no known competing financial interests or personal relationships that could have appeared to influence the work reported in this paper.

## Data Availability

Data will be made available on request.
